# Climate change and public health in Germany – A synthesis of options for action from the German status report on climate change and health 2023

**DOI:** 10.25646/11774

**Published:** 2023-11-29

**Authors:** Martin Mlinarić, Susanne Moebus, Cornelia Betsch, Elke Hertig, Judith Schröder, Julika Loss, Ramona Moosburger, Petra van Rüth, Sophie Gepp, Maike Voss, Wolfgang Straff, Tanja-Maria Kessel, Michaela Goecke, Andreas Matzarakis, Hildegard Niemann

**Affiliations:** 1 Robert Koch Institute, Department of Epidemiology and Health Monitoring, Berlin, Germany; 2 University of Duisburg-Essen, Germany, University Medicine Essen, Institute for Urban Public Health; 3 University of Erfurt, Germany, Institute for Planetary Health Behaviour; 4 Bernhard Nocht Institute for Tropical Medicine, Health Communication, Hamburg, Germany; 5 University of Augsburg, Germany, Faculty of Medicine; 6 German Environment Agency, Subject area I 1.6 KomPass – Climate Impacts and Adaptation, Dessau-Roßlau, Germany; 7 Centre for Planetary Health Policy, Berlin, Germany; 8 German Environment Agency, Subject area II 1.5 Environmental medicine and health assessment, Berlin, Germany; 9 Federal Centre for Health Education, Cologne, Germany; 10 German Meteorological Service, Research Centre Human Biometeorology, Freiburg, Germany

**Keywords:** CLIMATE PROTECTION, CLIMATE CHANGE ADAPTATION, PUBLIC HEALTH, CROSS-SECTORALITY, CO-BENEFITS, COMMUNICATION

## Abstract

**Background:**

This article represents the conclusion of the updated German status report on climate change and health, which was jointly written by authors from over 30 national institutions and organisations. The objectives are (a) to synthesise the options for action formulated in the report, (b) to combine them into clusters and guiding principles, (c) to address the success factors for implementation, and (d) to combine the options for action into target parameters.

**Methods:**

The options for action from the individual contributions of the status report were systematically recorded and categorised (n=236). Topical clusters were then formed with reference to Essential Public Health Functions, and options for action were assigned to them.

**Results:**

Eight topical clusters of options for action and ten guiding principles were identified. These can be summarised in four overarching meta-levels of action: (a) cross-sectorally coordinated structural and behavioural prevention, (b) monitoring, surveillance, and digitalisation (including early warning systems), (c) development of an ecologically sustainable and resilient public health system, and (d) information, communication, and participation. The main success factors for implementation are the design of governance, positive storytelling and risk communication, proactive management of conflicting goals, and a cross-sectoral co-benefit approach.

**Conclusions:**

Based on the status report, systematically compiled target parameters and concrete options for action are available for public health.

## 1. Introduction

Mainstreaming health, well-being, and equity into climate action and adaptation represents the greatest opportunity for a successful public health strategy in the early 21^st^ century [1]. The Paris Agreement (2015) commits the global community to effective climate action and solidarity with those most affected by climate impacts. This commitment involves necessary changes in technologies, infrastructures, consumption, culture, and policies that are interlinked and mutually reinforcing at the global, national, and local levels. In this regard, the Intergovernmental Panel on Climate Change (IPCC) argues at the end of the IPCC Sixth Assessment Report for the need for cross-sectoral and cross-system action to implement socially just climate change mitigation and adaptation for human well-being [2], advocating a holistic, interdisciplinary, and cross-sectoral approach to climate protection.

### 1.1 The context of climate change and health

Climate change affects human health, and thus the spread of communicable and non-communicable diseases (NCDs), as well as sociostructural dimensions through direct and indirect mechanisms [3, 4]. This concerns not only extreme weather events or the associated catastrophic events in Germany (e.g. floods in Rhineland-Palatinate and North Rhine-Westphalia in 2021), but also medical consequences that affect healthcare practice. For example, paediatricians are increasingly identifying important fields of action in the increased concentrations of air pollutants or pollen and exceptionally high ultraviolet (UV) radiation, as well as the associated diseases such as asthma and atopic dermatitis, but health education on these issues is lacking so far [5, 6]. The most recent report of the German Expert Council for Health and Care (SVR) from January 2023 considers measures for adaptation to the consequences of climate change to be a central task. In addition, the development and maintenance of resilient health systems is viewed as a global challenge [7]. A close link between the environment and healthcare sectors is also called for in the reports of the German Advisory Council on the Environment (SRU) [8] and the German Advisory Council on Global Change (WBGU) [9]. The SRU report identifies the urban environment as a hub for health-promoting and socioecological policies [8].

Both before and after the COVID-19 pandemic, many high-income countries, such as the Federal Republic of Germany, have faced public health challenges such as the morbidity and burden of disease due to NCDs, antimicrobial resistance (AMR), an ageing population, shortages of skilled workers, and health inequalities that place a heavy burden on healthcare systems, regardless of the impact of climate change [10]. Political will, multisectoral responsibilities, and systematic evaluation are needed to ensure the fulfilment of Essential Public Health Functions (EPHFs) such as monitoring and surveillance, equity of care, and governance [10]. The recommendations of the SRU and WBGU [8, 9] point in a similar direction.

The World Health Organization (WHO) developed a framework to increase the climate resilience of health systems [11, 12]. This framework addresses the areas of leadership and governance, climate and health financing, health workforce, vulnerability, capacity, and adaptation assessment, integrated risk monitoring and early warning systems, health and climate research, climate-resilient and sustainable technologies and infrastructures, the management of environmental determinants of health, climate-informed health programmes, and emergency preparedness and management [12].

The report of the SVR (2023) follows this framework as well as a version updated by WHO in 2015 [12] and sees a need for continuous preparation, learning, and adaptation processes in order to achieve resilience in the German healthcare system [7]. A distinction is made between preparation for crises and crisis management, and evidence-based policy advice and science communication are given a high-priority status [7]. The options for action from the status report on climate change and health described in this article are embedded in this context.

### 1.2 Objective and structure

The German status report on climate change and health was funded by the Federal Ministry of Health as part of the KlimGesundAkt project in view of the many challenges posed by the impacts of climate change on human health. For this purpose, more than 90 authors from over 30 national institutions, authorities, and (civil society) organisations wrote scientific articles and reviews on health-related and climate change-associated topics and derived recommendations from the gathered evidence.

**Figure 1:**
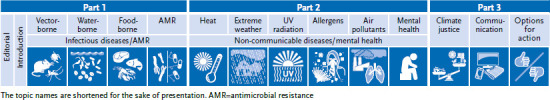
Topics of the German status report on climate change and health Source: Own representation

The status report consists of a total of 14 individual contributions in three parts (Figure 1) and aims to provide a scientific summary of the impacts of climate change on health. In addition, concrete options for action with regard to climate protection and adaptation measures are summarised in each individual article. In the first part, Hertig et al. [3] introduce the topic and the status report, followed by contributions discussing communicable infectious diseases (vector- and rodent-borne infections [13], waterborne infections and intoxications [14], foodborne infections and intoxications [15]) and AMR [16]. The second part features articles on NCDs caused by heat [17], extreme weather [18], UV radiation [19], allergens [20], or air pollutants [21]. In addition, possible influences of climate change on mental health are discussed [22]. The third and final part contains, in addition to this article, contributions on cross-cutting issues such as climate justice [23] and aspects of target group-oriented health communication [24]. Further communication of the results of the status report will also be carried out through (online) expert discussions with stakeholders as well as through mass media (press reports, TV, streaming, etc.) and digital channels (e.g. podcasts and videos on social media).

**Figure 2:**
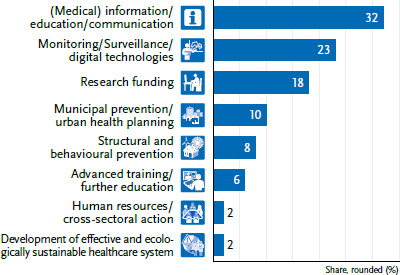
Topical clusters for n=62 options for action in the area of infectious diseases and antimicrobial resistance Source: Own representation

**Figure 3:**
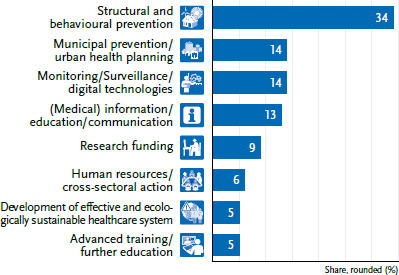
Topical clusters for n=174 options for action in the area of non-communicable diseases/mental health Source: Own representation

This article, the final individual contribution to the status report, aims to synthesise all the recommendations formulated in the other contributions, which are first classified into eight topical clusters (Figure 2 and Figure 3) and then into four meta-levels with subordinate guiding principles. After first describing the target groups of the options for action (Section 1.3 Target groups of the options for action), the second section explains the methodological procedure for the categorial clustering of the manifold options for action along the EPHFs (Section 2.1 Topical clusters). The categorised options for action, summarised into four meta-levels of action, as well as their guiding principles are explained in Section 2.2. Subsequently, the possible success factors for implementation are addressed. These include the design of governance, discursive risk communication and positive storytelling, proactively managing conflicting goals and resistance, and a cross-sectoral co-benefit approach. In Section 4 Target parameters for public health, the options for action are merged into short- to medium-term target parameters for public health. Conclusions and an outlook end the article.

### 1.3 Target groups of the options for action

The options for action from the individual articles of the status report are based on the current state of research and are intended to provide (federal state) authorities and municipal administrations with options for action and offer them support in implementing their climate and health-related goals. Due to German federalism and the subsidiarity between the federal, state, and local levels, only a rough framework of options can be presented here, which must be adapted contextually for each respective level. Expert discussions with stakeholders at the state and municipal levels conducted in parallel to the development of the status report indicated that some actors at these levels are already involved in climate change adaptation and mitigation measures.

It is the consensus of all the articles contributing to the status report that both climate change adaptation and mitigation measures as well as the protection of our health must be guaranteed. This can only be implemented by a cross-sectional approach in line with ‘Health in All Policies’ (HiAP) across the different sectors [8, 9, 25, 26]. This necessity is increasingly discussed in health science discourse by established and comparable integrative concepts such as One Health or Planetary Health [27].

The following target groups are considered priority actors for implementation:

1. Decision-makers from politics, federal/state ministries or agencies/authorities, cities, and municipalities, especially from the health, environmental, and urban planning areas
2. Facilities in the healthcare field (e.g. inpatient or outpatient services)
3. Stakeholders from relevant sectors (e.g. environment, construction, transport/mobility, urban health planning, and architecture)

In the further process of everyday implementation, the options for action are also directed at decision-makers in the public sector and in various settings (e.g. work, day care centres, schools, and sports/clubs) as well as the private sector (e.g. companies, organisations, and think tanks).

## 2. Options for action in the German status report on climate change and health

There is hardly any aspect of the environment and society that is not or will not be affected by climate change, at least indirectly. In many areas, comprehensive transformations are on the agenda – both to achieve climate goals and to minimise the consequences of climate change. The first steps have already been taken in Germany. The Federal Centre for Health Education (BZgA) has begun to summarise climate-related health risks, with a focus on heat and UV protection, giving concrete recommendations (www.klima-mensch-gesundheit.de); other topics such as allergies and vector-borne diseases will be added in the near future.

At the 93rd conference of German federal health ministers in September 2020, it was agreed that heat-health action plans (HHAPs) should be prepared by 2025, primarily at the municipal level, taking into account local capacities [28]. However, the Association of German Cities indicated in a discussion paper from May 2023 that the implementation of such HHAPs by 2025 would not be feasible due to scarce resources in many municipalities without advice, funding, and support from the federal states (‘Länder’) and the federal government [29].

Heat protection and related communication are often the first measures of climate change-related health policy implemented at the municipal level, and one of the priority fields of action in the health sector. In German municipal administrative action, the implementation of such measures is often complete (41%) or in progress (22%). In contrast, the monitoring of heat-related mortality or morbidity has only been implemented in 8% of the surveyed federal states, districts, cities, and municipalities [30]. The German Environment Agency (UBA) concludes in a recent analysis that many municipalities in Germany are in the development or establishment stage of an HHAP, but are rarely in a position to prioritise this [31].

The options for action from the status report on climate change and health listed below support the processes that have been initiated. However, they also imply the need to think beyond heat adaptation and to provide the necessary resources.

### 2.1 Topical clusters

The contributions to the status report on the impact of climate change on communicable diseases and NCDs and mental health formulated 236 recommendations at very different levels, with heterogeneous challenges and degrees of complexity. Based on a category system that was collaboratively reviewed (by MM and HN), eight topical clusters were developed from 62 individual options for action from the section on infectious diseases (Figure 2) and from 174 individual options for action from the section on NCDs and mental health (Figure 3).

These topical clusters were, as far as possible, deductively assigned following the national EPHFs of the German Zukunftsforum Public Health (future forum on public health) [32] as well as the EPHFs which were systematically related to environmental and climate aspects for the first time by the WHO Pan-American Regional Office [33] and referred to in the introductory article of the status report by Hertig et al. [3]. Where this could not be applied, new inductive categories were formed. The reference to the EPHFs serves to provide a clear synthesis of the recommendations from the individual articles on infectious diseases, NCDs, and mental health.

As depicted in Figure 2, contributions on infectious diseases and AMR tend to prioritise (medical) information, communication, and education needs (32%), whereas the articles on NCDs/mental health focus more strongly on structural and behavioural prevention (34%), including in a municipal setting (14%) (Figure 3). This includes, for example, municipal HHAPs and climate protection through green hospitals or diverse urban health planning. Recommendations on monitoring and surveillance, including digital technologies (e.g. artificial intelligence (AI) for imaging) and early warning systems, are also frequently mentioned. Overall, the need for further research is given a high priority. For example, further basic research is needed on the effects of climate change on certain vectors [13], the occurrence of pathogens in food and water [14, 15], AMR [16], or the combined effects of air pollutants and temperatures [17, 21].

In general, the recommendations for action are in line with the findings of the European Environment Agency [34], which also identified that interventions have a focus on monitoring and surveillance (including the establishment of early warning systems), communication campaigns to raise awareness among the general population, and the promotion of further research for the European region. Cross-cutting issues such as social inequalities and climate justice are included in many of the cluster dimensions mentioned above. Bolte et al. [23] devote a separate article to this important topic in this status report.

### 2.2 Meta-levels of action and guiding principles

In the following sections, examples of the options for action identified in the eight topical clusters are described and specified by formulating guiding principles. For the detailed options for action, we refer to the original articles on the respective specific subjects. Four basic meta-levels of action can be derived from the eight topical clusters presented (Table 1).

#### Cross-sectorally coordinated structural and behavioural prevention

For prevention to be successful, structural and behavioural changes must be optimally coordinated [35]. Thus, educating and informing the population should go hand in hand with the creation of health-promoting and climate-friendly structures and conditions [26]. Structural prevention measures aim at generating a sustainable and effective change in social, structural, and cultural conditions for the best possible opportunities for all to maintain and improve their health. These include, above all, planning, technical, and regulatory measures to combat climate change-related health risks.

##### Guiding principle: ‘Implementing cross-sectoral planning processes’

How can cross-sectoral planning processes be implemented? One example in Germany includes sub-national HHAPs [36, 37]. Information, advice, and guidelines are provided at the federal level (e.g. by UBA, BZgA, and the Federal Institute for Occupational Safety and Health). Tools are available from universities (Fulda [31, 38]) and alliances (Berlin [39]) as well as through structured state-wide HHAPs (Hesse [40]), for whose implementation municipalities are responsible. Funding can be provided by existing local budgetary resources or through projects. Civil society participation should also be considered in the planning stage. Various elements and steps are necessary in the development of HHAPs [8]. For example, networking will help in clarifying the roles and responsibilities of various local offices and actors at an early stage [31]. In HHAPs, thermal stress should be addressed while, at the same time, considering protective measures against UV radiation, as well as UV radiation-associated air pollution, such as ground-level ozone, air pollutant, and allergen loads [17, 36, 37]. When developing holistic climate adaptation plans, the potentially seasonal occurrence of pathogenic viruses in water bodies should also be considered in regions rich in rivers and lakes [14]. According to Winklmayr et al. [17], continuous monitoring and evaluation is necessary to determine the effectiveness of HHAPs and integrated measures. The example of HHAPs shows that cooperation between the different sectors involved in climate adaptation (or climate change mitigation) planning processes is essential.

##### Guiding principle: ‘Implementing urban (health) planning measures’

Urban planning measures with regard to climate adaptation, such as increasing the number of green and blue spaces to minimise heat island effects, are highlighted in the report’s contributions relating to heat, UV radiation, and air pollutants [17, 19, 21]. Building insulation and better ventilation concepts should also be considered [19]. Green mobility, including the transformation of road traffic space and energy-efficient re-structuring measures, is a big component of climate protection. In each of these areas, it is important from a public health perspective to pay attention to health equity to promote environmental justice [23, 41]. Vulnerable groups, such as people with respiratory and cardiovascular diseases in urban heat islands, who may live in disadvantaged neighbourhoods, are particularly exposed [8, 9, 20].

Urban green spaces, such as parks, road-side trees, and green roofs, form recreational spaces. Cold urban spaces and cooler buildings through green facades improve air quality and have a positive effect on well-being [3, 20]. If walking and cycling paths in the city are expanded, and made safer and greener, travelling on foot or by bicycle instead of by car (active transport) becomes more attractive. This can reduce CO_2_ emissions while at the same time promoting health through the promotion of physical activity and reduced particulate matter pollution (so-called co-benefits between climate and health protection) [2].

Adaptation measures take place in complex systems. This can lead to unexpected and unintended outcomes within subsystems. The creation of blue infrastructure can reduce the air temperature, but also promote breeding sites for mosquitoes and thus, potentially, vector-borne diseases. Planting trees in road-side areas can provide shade, but can also impair the vertical mixing of the air and thus promote the ground-level accumulation of air pollutants and, depending on the tree species, increase allergy exposure. These examples of trade-off effects already show the numerous interactions between climate, environment, and health (cf. conflicting goals in Section 3.3 Proactively managing conflicting goals and resistance) and clearly imply the need for cross-sectoral and transdisciplinary cooperation in the development and implementation of adaptation measures.

##### Guiding principle: ‘Using governance for structural prevention’

Many individual contributions in the status report recommend cross-sectoral structural prevention as well as even more far-reaching policies at the (sub-)national level. For example, Breitner-Busch et al. [21] call for a common strategy to combat air pollutants, temperature, pollen, and UV exposure in order to achieve effective action. Regarding air pollutants, reduced limit values (including for particulate matter and nitrogen dioxide) in the European Union (EU) are an essential step towards improving air quality and reducing the burden of disease in Europe [21]. The article by Baldermann et al. [19] also advocates for behavioural and structural prevention measures to prevent UV-related diseases. The legal framework for this includes the Prevention Act, the Early Cancer Detection and Registry Act, and the ‘Patientenrechtegesetz’ (Act on Patients’ Rights) [19].

##### Guiding principle: ‘Using existing tools in development and planning’

Tools and digital resources for municipal decision-makers must be communicated and further developed in a participatory manner – for example, in formats employed by the German Zukunftsforum Public Health [32, 42], the Federal Association of Physicians of German Public Health Departments, or the German Association of Cities and German County Association. Examples of such tools include the cross-sectoral recommendations of the German Environment Agency for urban health [43] or various recommendations for the preparation of HHAPs [8, 30, 31, 36-38, 40]. An incomplete list of existing tools regarding heat, climate communication and monitoring, existing digital platforms, networks, and contact points can be found in the Info box. The use of such resources and collaborative structures for knowledge transfer is recommended in the literature [26].

**Info box Selection of available tools (as of October 2023), some in German**

**Heat, heat-health action plans (HHAPs), and climate adaptation**

‣ Governmental recommendations for action for the preparation of heat action plans to protect human health
‣ Working aid for the development and implementation of an HHAP for cities and municipalities from Fulda University of Applied Sciences
‣ Hessian HHAP of the Hessian Ministry for Social Affairs and Integration
‣ The Ludwig Maximilian University of Munich’s heat service for municipalities
‣ Information portal of the German Alliance on Climate Change and Health

**Climate communication and climate change-related health data**

‣ Robert Koch Institute: www.rki.de/klimawandel | http://www.rki.de/climatereport
‣ Federal Centre for Health Education: Climate-Human-Health and StadtRaumMonitor
‣ German Alliance on Climate Change and Health
‣ Centre for Planetary Health Policy
‣ Helmholtz Climate Initiative
‣ Explorer of the Planetary Health Action Survey (PACE)
‣ European Climate and Health Observatory
‣ Geoportal
‣ Information system of the Federal Health Reporting
‣ Mosquito Atlas
‣ Topical data and information sources: e.g. UV Index, allergy information service, National Expert Group ‘Mosquitoes as Vectors of Disease Agents’, KABS e.V. (Asian tiger mosquito)

**Digital platforms, networks, and contact points**

‣ Agora exchange platform for public health agencies
‣ The German Environment Agency’s ‘action database’
‣ German Climate Preparedness Portal
‣ Federal information centre for climate adaptation
‣ Service point for municipal climate protection of the German Institute of Urban Affairs
‣ Association of German Cities and German County Association
‣ Inforo – Portal for professional exchange

##### Guiding principle: ‘Promoting behavioural prevention through structures’

Several recommendations for climate-sensitive and climate-protective health behaviour as well as climate-informed health promotion can be found in the individual contributions of the status report. Structural prevention should encourage the intended behaviour [17, 19, 21, 22], for example by financially supporting the wide establishment of health promotion measures. Even the protection of mental health may be strengthened by sustainable mitigation (climate protection) and adaptation measures [21].

The following examples do not claim to be exhaustive, but serve to illustrate beneficial frameworks and structures. Further education and training of health professionals, such as general practitioners, is suggested for increasing the likelihood of behavioural prevention and health promotion, for example in the area of vector- and rodent-borne diseases [13]. Measures for information and knowledge transfer are also required. In the context of waterborne infections, it would be helpful to increase awareness of *Legionella* in water supply systems, but also to adapt bathing behaviour in shallow waters, such as ‘bodden’ waters (briny coastal lagoons), or to avoid bathing in saline waters with open wounds [14]. In the area of UV radiation, certain behavioural habits regarding individual UV exposure and knowledge of the UV index would be helpful [19], and could be greatly supported by UV index displays in public spaces (e.g. open-air swimming pools, city squares, or waiting areas for local and long-distance public transport). The creation of shaded areas and the adaptation of workday routines would additionally support healthy, climate-sensitive behaviour [19]. It is also important to avoid health risks caused by air pollutants or heat. In particular, people with pre-existing diseases should avoid exertion during the midday and afternoon hours when heat and ozone concentrations are high [17, 21]. However, a higher acceptance of heat breaks (siestas) would be required, e.g. in occupational contexts.

#### Monitoring, surveillance, and digitalisation (including early warning systems)

As part of the German Strategy for Adaptation to Climate Change (DAS) of 2008 [44], the Federal Government has published a monitoring report on the DAS every four years since 2015, in which the development of climate impacts and adaptation in 17 fields of action is monitored based on several indicators. One field of action is human health. The new Climate Adaptation Act provides a legal framework for the development of a federal climate adaptation strategy with measurable objectives, indicators, and measures. Health is one of the eight clusters named. All federal ministries and the authorities within their remit are involved in the work on the DAS, as are the federal states. In 2015 and 2019, monitoring reports were published by the federal government that present climate change-related indicators in time series [45]. In order to assess future risks, the Climate Impact and Risk Assessment (KWRA) is regularly carried out by a cooperation of 28 federal authorities [46]. In the KWRA 2021, climate risks were identified for different time horizons: the present, as well as the middle, and end of the century. Heat, UV exposure, and pollen were identified as in particular need of action [46, 47]. This assessment is based on various assumptions about future climatic and socioeconomic developments.

##### Guiding principle: ‘Strengthen monitoring and surveillance’

Through monitoring and surveillance, appropriate indicators can be used to identify and prioritise knowledge gaps in relation to the impact of climate change on health, as well as with regard to progress through climate protection and the mitigation of climate impacts. Increasing heat stress and the rising number of heat events will be of central relevance in the context of the densification of inner cities and the high population density in urban agglomerations [17]. In the DAS monitoring, these issues are addressed by indicators for heat stress and estimated heat-related mortality. In addition, heat stress in cities and heat island effects during summer are represented by data from the German Meteorological Service (DWD). Data on emergency admissions in municipal hospitals and associated mortality monitoring could add small-scale information [17]. In the area of food safety, the identification and investigation of the geographical distribution of toxin-producing organisms may be of crucial importance for the implementation of appropriate preventive and control measures before harvesting or during the distribution and sale of seafood [15].

Global warming is extending the pollen season and increasing the habitat for some allergenic plants. This can be assessed by monitoring pollen counts (e.g. with indicators from the DAS monitoring report), allergies, and sensitisation to specific allergens [20]. Due to climate change-related changes in the factors influencing UV radiation exposure, this exposure is changing in Germany, which may influence the UV-related risk of disease. This can be assessed by monitoring UV irradiance, annual UV dose, and UV-related health outcomes [19]. Air pollutant concentrations are also affected by weather and atmospheric conditions. For example, concentrations of ground-level ozone could increase, requiring air pollutant monitoring [21]. An air monitoring network is already being operated by the German Environment Agency in cooperation with the federal states.

Increasing temperatures favour the growth of microorganisms and algae. Non-cholera *Vibrio*, bacteria which occur in brackish water and seawater, can cause fatal infections in vulnerable individuals with pre-existing diseases, with the potential for infection increasing as the oceans warm [14]. Efficient methods will have to be developed both to detect the (often new) pathogens (viruses) and to eliminate them. Disease vectors such as (tiger) mosquitoes, ticks, and bank voles are influenced by climate change in their distribution, frequency, and activity. This may lead to the increased spread of (non-)indigenous vectors and diseases, such as tick-borne encephalitis (TBE), Lyme disease, dengue fever, as well as chikungunya and hantavirus [13]. With regard to TBE alone, 46 new risk areas were identified in southern and central Germany in the period between 2007 and 2022 [13]. In the context of vector- and rodent-borne infectious diseases, it is advisable to continuously expand existing monitoring and surveillance methods as well as research on the topic and to adapt them to changing environmental conditions [13].

The DAS Monitoring Report 2023 introduces an indicator presenting data from the Citizen Science Project ‘Mosquito Atlas’, which was launched by the Leibniz Centre for Agricultural Landscape Research and the Friedrich-Loeffler-Institut in 2012. The mosquito atlas is a part of scientific mosquito surveillance and allows an overview of the distribution of invasive species, while also making the population aware of the problematic introduction and spread of invasive mosquitoes. However, these data do not provide systematic information on the spread of invasive mosquito species.

More frequent extreme weather events, such as the low-pressure area ‘Bernd’ in July 2021 and the resulting floods and landslides, are increasing the demands on the healthcare system [18]. Overall, the number of weather-related cases of illness will most likely rise, placing high demands on the healthcare system and the reliability of infrastructures (e.g. Federal Agency for Technical Relief, disaster protection). On the adaptation side, the use of some warning and information systems has been reported so far, such as the DWD’s heat-health warning system, which has been in place since 2005, and the information on pollen pollution provided by the German Pollen Information Service Foundation and DWD [17, 20].

So far, the results of the DAS monitoring are classified by trend assessments. Currently, the federal ministries are working on the further development of the DAS into a preventive climate adaptation strategy with measurable objectives. Parallel to this process, civil society is also striving to report the monitoring more precisely and more closely for individual national countries in line with the Lancet Countdown in Europe [1], as it has so far only been a matter of modelling and rough projections. The Lancet Countdown Report (2022) also recommends national indicators for monitoring climate change-related health and environmental policies [1].

##### Guiding principle: ‘Using digital technologies for climate adaptation and climate protection’

In connection with modern monitoring and surveillance as well as early warning systems, digital technologies, which represent the infrastructural basis, should also be mentioned. Practical examples include the development of geographic information system-based modelling programmes for the visualisation of UV exposure for urban and building planning as well as landscape architecture for the creation of UV-reduced outdoor areas [19]. In pollen monitoring, the available repertoire of methods is expanding due to the technical developments of recent years (such as digital or AI-based image recognition processes) to include options for digital pollen recognition [20]. Digital technologies, such as blockchain or radio frequency identification device tags, can support sustainable and climate-protective development in the seafood industry [15]. However, the use of automated techniques can require high acquisition and operating costs, as well as a skilled workforce. For extreme weather events such as heavy rain, storms, and heatwaves, early warning systems and timely risk communication can minimise the negative health outcomes. Digital technologies, such as AI or warning apps, can play an important role here as part of a mix of warning systems (including sirens or warning messages via mobile phone) [18].

#### Development of an ecologically sustainable and resilient public health system

The current report of the SVR considers climate change to be a central stressor and an essential touchstone for increasing resilience in the healthcare system [7]. Following WHO (2015), the report sees approaches for increasing resilience in the fields of governance, financing, healthcare personnel, integrated risk monitoring and early warning systems, in addition to research, as well as in climate-resilient and ecologically sustainable (healthcare) technologies, programmes, and infrastructures [7, 12]. The area of governance and financing concerns, for example, the level of (self-governing) municipalities and districts in the German federal states. This administrative level is essential for the implementation of health-related climate adaptation and protection policies [48].

##### Guiding principle: ‘Making the healthcare system sustainable and climate-friendly’

The ‘development of an effective and ecologically sustainable healthcare system’ cluster identified in the status report’s recommendations (Figure 2 and Figure 3) includes the aspects of environmental protection and sustainability. Breitner-Busch et al. [21], for example, state that the German healthcare sector is responsible for approximately 6% of Germany’s overall greenhouse gas emissions. Therefore, the healthcare system is called upon to ‘permanently minimise its greenhouse gas emissions as a climate protection and air pollution control measure while maintaining the same quality of basic care and high-quality standard of the services provided’ [21, P. 115].

Structural and functional investments and changes leading to climate-neutral hospitals (green hospitals) or medical practices are mentioned, such as innovative air conditioning. Such structural investments in adaptive facilities are expensive and require financial support [13]. Large organisations in the healthcare sector, such as inpatient healthcare facilities that run 24/7, consume enormous amounts of energy [21]. Consequently, energy retrofits to make hospitals and medical practices climate-adapted and climate-neutral are an indispensable mitigation measure. The care of patients has top priority and ‘in view of increasing extreme heat events, adaptation measures for structural and air-conditioning renovation, shading, passive building cooling and, if medically necessary, individual room air-conditioning powered by renewable energies must be implemented’ [21, P. 114]. Concrete measures in this direction have not yet been taken [21].

#### Information, communication, and participation

The last topical cluster on ‘(medical) information, education, and communication’ is relevant throughout all the topics, as the effects on infectious diseases, NCDs, or mental health are often insufficiently known or underestimated in many relevant target groups, as shown by Lehrer et al. [24] in another article in this status report. One question in this context is the extent to which patients are given up-to-date, evidence-based information by doctors with regard to UV, allergens, or the interaction between heat and certain medications [17, 19, 20].

##### Guiding principle: ‘Sharing knowledge and educating through participation’

A crucial factor for the implementation of an ecologically sustainable and resilient public health system is the continuing education of health professionals. The central importance of (continuing) education and training for health professionals on the clinical consequences of climate change is mentioned in various contributions to the status report [13, 17, 19, 21, 22]. The further training of professionals in human and veterinary medical practices or public health service facilities is necessary with regard to behavioural prevention and health promotion in the field of vector-borne diseases [13]. The need for the additional training and education of healthcare personnel is also present in the area of heat or UV protection in the employment, social, education, and (health)care sectors [17, 19]. Sustainability and environmentally friendly behaviour at the workplace also rely on the mindful use of disposable products and consumables in everyday care. The content of nursing and therapeutic training, medical school curricula, and further training regulations of the medical associations should be adapted to include the health impacts of climate change. The examination regulations of the German National Institute for State Examinations in Medicine, Pharmacy and Psychotherapy should also include relevant learning objectives.

As healthcare workers are among the most trusted groups in society, they should be supported in providing communication (see Section 3.2 Positive storytelling and risk communication) [49–52]. Communication about climate change could be included in medical consultations regarding heat, allergens, UV, and mental health [17, 19, 20, 22]. The idea of participation could receive a higher priority in planning, development, and implementation for climate change adaptation (e.g. HHAP) or mitigation in public (healthcare) facilities [22]. This could be achieved by the establishment of forums and open spaces in which representatives of socially disadvantaged and marginalised groups in particular are allowed to discuss and decide on measures [23]. Everyday settings such as day care centres and schools, voluntary associations, and sports clubs represent further areas with the potential for more participation. Staff should be trained regarding the health impacts of climate change, adaptation, and protection and be involved in implementation in a participatory manner.

##### Guiding principle: ‘Promoting target group-oriented awareness raising and social equity’

In order to raise awareness for the health impacts of climate change and to support adaptation processes, target group-specific communication, which is understood by and practically relevant to the respective target group, is crucial. In the articles concerning infectious diseases, information and education measures are proposed for different target groups:

1. Information and education campaigns to raise awareness and preventive measures on the risk of infection (including mosquito reproduction sites and the spread of new mosquito species, as well as potentially infectious water contacts through bacteria, vector- and water-borne infections) [13, 14]
2. The education of target groups (e.g. people who work in the forest or spend their leisure time there) and medical professionals about Lyme disease [13]
3. Seasonal and targeted communication on the risk posed by hantavirus infections [13]
4. Kitchen hygiene measures to protect against foodborne intoxications [15]
5. Risk communication and warning the population about the health consequences of infectious diseases as a potential result of extreme weather events [3, 18]

In the area of NCDs, the article on extreme weather events also mentions numerous established early warning systems, such as heavy rain hazard maps, as well as the forest fire hazard index and grassland fire index [18]. UV exposure should also be integrated into early warning systems on a permanent basis (as is already the case with the heat-health warning system) [19]. Information and education on UV protection measures for children and adolescents in day care centres and schools, which also involves their parents, is necessary [19]. This requires the creation and distribution of target group-oriented information material. Graphical and easily understandable information materials can be helpful for socially disadvantaged or vulnerable groups [53]. In Germany, it would be advisable to include professional expertise from the Collaborative Network for Equity in Health.

## 3. Discussion of the success factors for implementation

Which success factors contribute to the successful implementation of the addressed options for action? In the following section we discuss four factors identified as promising in the literature and in individual contributions to the status report:

1. Design of governance
2. Positive storytelling and risk communication
3. Proactively managing conflicting goals and resistance
4. Cross-sectoral co-benefit approach

### 3.1 Design of governance

The bottom line of the topical clusters of recommendation – in agreement with the results of the European Climate and Health Observatory – is to achieve a policy mix of coordinated structural and behavioural prevention [34], which has been applied in other fields of public health or NCD prevention for decades (e.g. tobacco control, human immunodeficiency virus, or diabetes prevention) [35]. Consequently, the complex challenges regarding the environment, climate, and health cannot be met without adequate governance and the incentive-driven (re)design of legal frameworks [8, 9, 17, 19, 25]. This concerns legislation and implementation issues in vertical governance at all levels (EU, federal, state, and local), such as the implementation of HiAP or other environmental and sustainability strategies [8, 9]. Heat protection and the topic of climate change and health should be implemented as a mandatory task in the public health laws of all German federal states. The observation and assessment of the effects of climate on human health as well as the measures within the federal states’ legal area of responsibility, including heat protection, could be anchored in the so-called health-service laws and regulations of the federal states. Furthermore, although air quality standards are in place at national and EU level, further measures are needed to lower the levels of particulate matter and nitrogen dioxide according to current WHO recommendations [8, 21]. The first important steps towards more ambitious limit values for air pollutants based on the WHO guidelines – in line with the recommendations of the Environmental Public Health Commission [54] – were adopted by the European Parliament in September 2023. Finally, the pillars of funding and resource allocation are central to ensuring cross-sectoral action in the sense of HiAP-informed health prevention and promotion [7–9, 17, 19–22]. Cross-sectoral cooperation or advocacy coalitions are essential for successful health promotion and prevention work. In order to promote the acceptance of the measures, active and independent education and scientific communication about the connection between climate change and health are indispensable [55]. Current initiatives by the German Federal Ministry of Health to implement a coordinated national heat-health protection plan for Germany take these aspects into consideration [56].

### 3.2 Positive storytelling and risk communication

Health-related science communication should take up and actively use existing findings and evidence from behavioural and social sciences [55]. Academic and mass media discourses on climate change are partly characterised by negative images (e.g. of droughts or severe weather disasters) and societal fears of loss [57, 58]. However, in addition to the negative health consequences widely discussed in this report, climate change adaptation and mitigation also offer considerable opportunities or chances for human well-being [59]. It is important to present the risks adequately, but at the same time also to mention opportunities and to communicate the options for action for individuals and society through positive storytelling.

The healthcare sector, as an area affected by the consequences of climate change, can lead the way in a bottom-up process and actively use the high level of trust placed in healthcare workers such as doctors and nurses in communication [49–52]. The design of concrete measures in specific settings should be evidence- and data-based and oriented towards the target groups concerned [60]. Arguments that emphasise the health benefits and opportunities for many stakeholders can help ensure that climate policy measures receive improved public and political resonance [49, 61]. Emphasis should be placed on the additional health benefits of climate protection measures, i.e. the discussion of co-benefits (see Section 3.4 Cross-sectoral co-benefit approach) with the positive health consequences and opportunities offered by climate-friendly structural and behavioural prevention, e.g. in the areas of transport, nutrition, and air quality [48, 59].

In risk and crisis communication, the focus is primarily on health education and information on the adverse health effects of climate change on human health. This may be necessary in the case of extreme weather events, heat, air pollution, or UV exposure and their direct adverse health consequences [62], in order to increase personal risk perception. However, it is essential that ways to avert the risks and thus increase self-efficacy are shown as well. Communication exclusively based on factual risks can lead to climate fatigue and reactance [24].

In order to push for action on health-related climate change mitigation and adaptation, studies argue for the use of positive images of the future and motivational storytelling [63]. The Persuasive Hope Theory assumes that hope appeals can increase the experience of self-efficacy and the readiness to act against climate change [64], but there is a dearth of studies proving causality. Nevertheless, when targeting the general population, it may make sense to avoid fear appeals and instead address emotional topics (e.g. the future and the well-being of one’s own children) [63, 65].

Access to as well as the understanding and acceptance of health information can be barriers in health communication related to prevention and health promotion [60]. To address target groups, these must first be identified. According to Lehrer et al. [24], younger people, men, people with lower levels of education, and people from smaller or rural communities in Germany are relevant target groups for climate change-related communication, as they have a greater potential to increase their readiness to act [24]. The majority of the public (at least 6 out of 10 people) support and accept climate protection measures both internationally [66, 67] as well as in Germany.

In line with these findings, other surveys show that public support for the ‘Energiewende’ (transition to a low-carbon energy supply) in Germany is at approximately 70%, but that 50% criticise its implementation [68]. A German-language online survey by the University of Erfurt (Planetary Health Action Survey, PACE) shows that public support for measures is systematically underestimated. Although the majority support climate protection measures, societal approval is subjectively underestimated by individuals. The situation is similar for concrete behaviours, such as the willingness to reduce the amount of meat in one’s diet [69].

According to the PACE study, the climate policy guidelines developed by the Citizens’ Assembly on Climate, a body consisting of 160 citizens (including statements, in German, such as ‘The future of the economy must be climate-neutral’) are readily accepted by two thirds of the respondents in Germany [70, 71]. However, knowledge or acceptance are not yet leading to behavioural change. Stimulating behavioural change through communication strategies alone is difficult and it should not be expected that the ‘correct’ formulations will achieve great effects. Communication measures should always be integrated into structural interventions or measures. Behavioural changes also face structural barriers in an individual’s living environment. A rapid change in attitudes, beliefs, values, and norms is subject to clear sociological and psychological limits [67, 72].

Surveys from the United States, Canada, and Germany show that seven out of ten people are concerned or alarmed about climate change, while one third are indifferent, doubtful, or even opposed to the issue [67, 70, 72]. Paradoxes within lifestyles (e.g. environmental and sustainability orientation vs. the desire for long-distance travel) or conflicting values, habits (e.g. meat consumption), and structural frameworks often stand in the way of individual climate protection behaviour even among sensitised people [71]. This is the case, for example, when balancing daily travel (to work) and family obligations (e.g. care work). Therefore, it is particularly important to facilitate climate-friendly behaviour through structural measures in the environment.

### 3.3 Proactively managing conflicting goals and resistance

Through differentiated planning and targeted action, one can proactively counter possible conflicting goals or resistance between sectors and interest groups. It can therefore make sense in health-related climate communication to connect to the interests of the respective target group [61, 73]. Consequently, it is of paramount importance to resolve conflicting goals and interests between competing and conflicting sectors, disciplines, and societal units (e.g. politics, science, civil society, or the economy) in favour of positive synergies and reminders of co-benefits [59]. Examples of cross-sectoral co-benefits discussed in the literature relate to issues such as air quality, food, and energy [74, 75]. Participatory approaches and cross-sectoral planning and decision-making formats are recommended [51, 76].

Such negotiation processes in complex societies are neither trivial nor free of conflict due to the different interests, diverse trade-off effects, and various stakeholders from science, civil society, politics, and business [77]. But even if the participatory negotiation of compromises and solutions is time-consuming and resource-intensive, such an approach – especially in times of often ideologically motivated climate and science scepticism [61] – creates more trust and legitimacy in the long run [76]. The participatory involvement of target groups in the development of risk communication strategies has proven to be a success factor e.g. in the development of disaster response plans [51]. These participatory negotiation processes dealing with trade-off effects (or conflicting goals) that arise at the municipal level require the establishment of exchange forums that support and accompany the processes using a cross-sectoral and evidence-based approach.

With regard to the healthcare sector, it is important to find a concrete and tailored set of measures for the daily relevant processes in this societal subsystem, and to involve ‘climate champions’, equipped with a high level of trust and credibility, in a participatory manner [48–50]. One central consideration should be to strategically appeal to the set of values, interests, and relevant topics of a target group, such as managing directors or hospital administrations in the context of establishing green hospitals [50, 57, 63, 66, 67]. Decision-makers in politics and administration should be able to recognise a concrete, cost-effective, and profitable effect in their area of responsibility (e.g. hospital planning or the health department). It is crucial to reach target groups with communication offers and dialogue formats in their settings, to initiate self-reflection processes, and thus to support possible changes in attitudes and behaviours.

### 3.4 Cross-sectoral co-benefit approach

Many climate protection measures related to nutrition and physical activity have positive economic, social, or climate policy consequences while also leading to health-promoting effects for individuals or population groups [78–82]. Such win-win constellations and co-benefits between climate-friendly and health-promoting actions were described in the Lancet in 2017 [59]. Health- and climate-related co-benefits are primarily those (positive) health effects that occur as a consequence of mitigation measures [83]. Frequently cited examples occur in the areas of physical activity and nutrition [79–82].

For example, the transport and agricultural sectors in Germany are responsible for a significant share of greenhouse gas emissions. If people walk or cycle more instead of using the car and consume fewer animal products, this does more than reduce greenhouse gases. Both behaviours – active mobility and the reduced consumption of (red) meat – also reduce the risk of various chronic diseases such as cancer or cardiovascular diseases [78–82]. In addition, there are health effects at the population level as well as environmental and climate effects if active mobility actually replaces car journeys: air pollution, exacerbated by cars through nitrogen dioxide emissions and particulate matter from combustion engines, brakes, and tyres, is reduced. Since the administration of antibiotics is a daily routine in factory farming, many antibiotic resistances develop in that area; resistant germs can also become dangerous for humans [84]. In the status report contribution on AMR [16], the reduced consumption of animal products is mentioned as an important strategy to reduce antibiotic resistance in livestock and thus also in humans, leading to a further health-related benefit.

Further examples are listed in the IPCC reports [85], for example with regard to effective mitigation. Building renovations and switching to renewable energies not only reduce greenhouse gas emissions, but also improve air quality (e.g. indoors) and thermal comfort by reducing heat effects. A health-related co-benefit approach can therefore be a tool to accompany the implementation of climate measures and to create increased acceptance [59, 86]. Based on public and political debates about win-win solutions, the co-benefit approach has found increasing resonance in political climate discourse [83].

The consideration of co-benefits also includes thinking about measures for mitigating climate change on the one hand and for adapting to climate change on the other. If, for example, people increasingly travel actively instead of by car, they may also be more exposed to climatic stresses such as heat and UV radiation. Precautions to improve the microclimate along walking and cycling routes should therefore be taken, such as using suitable building materials or increasing green and blue spaces and shade [87].

The examples show that it is not sufficient to take short-term remedial measures to alleviate climate change impacts; structural changes also need to be made through the cooperation of the public health sector with other sectors. A broad understanding of health-promoting environments is important. According to WHO, we need to ‘rethink the way we live, work, produce, consume and govern (…). The health sector needs to play a new role to drive this transformation’ [86, P. 7].

Health-related co-benefits can be a key factor in a successful transformation. Public health is thus not only an important element in shaping socioecological change, but must also actively engage as a driver in the transformation process [85].

Emphasising the additional health benefits of climate protection measures also makes health-promoting effects more visible and tangible for many people than climatic ones, which are often longer-term, appear diffuse, and tend to be located in other regions of the world. Indications of individual additional health benefits can thus counteract the feeling of spatiotemporal distancing from the consequences of climate change [88].

Actors in the field of climate and environment at the (inter)national and municipal levels do not yet systematically prioritise health as a factor or driver. Health as a driver does not appear in the German Climate Change Act, and the German Climate Action Plan 2050 only mentions health in connection with construction projects. The German government’s DAS, on the other hand, has included human health as a separate field of action. The indicators chosen in this field include, for example, heat-related mortality, allergenic plants, exotic insects, cyanobacteria, and pollen [45]. However, the focus here is clearly on health risks that can often only be countered in a behavioural way, instead of co-benefits accompanied by structural prevention.

Due to the multitude of co-benefits for health, climate, and environment, the implementation of climate change mitigation and adaptation measures can benefit from the public health perspective and existing public health structures; at the same time, the health sector can benefit from experiences and activities in the environmental and climate field.

Overall, it can be concluded that, with regard to health-related co-benefits, there is potential in climate change mitigation and adaptation to reduce the gap between knowledge about the impacts of climate change and taking appropriate action. The health perspective can serve as an argument to underpin motivation for action at both policy and individual levels and should be increasingly used [59]. It can also serve as a guide for sectoral cooperation between the health, environmental, and urban planning sectors for structural action. New research may also help to identify further co-benefits in the complex interactions of climate, environment, health, welfare, and economics [59].

## 4. Target parameters for public health

Climate change poses major challenges for societies worldwide. One of the major challenges in climate change mitigation and adaptation is the inequality dimension (see Bolte et al. [23]). Although this report has a clear focus on Germany, the challenge of climate change and inequality cannot be tackled without a global perspective. The poorer half of the world’s population will remain far below a level of greenhouse gas emissions compatible with the 1.5°C limit in 2030. The world’s richest ten percent, on the other hand, will exceed this level ninefold by the same date [89].

This underlines the necessary commitment of a globally influential high-income country, such as the Federal Republic of Germany, to engage in this process as a key climate actor. Short- to medium-term measures should prioritise holistic climate protection alongside locally necessary adaptations. This must be done in the context of interdepartmental and cross-sectoral coordination. From a health, science, and long-term economic perspective, holistic climate protection for people’s well-being and health should be prioritised. For the sake of an interdisciplinary and cross-sectoral approach, it is important to name conflicting goals between sectors or disciplines (e.g. health and construction or urban planning), in politics, administration, and civil society, and to resolve them as quickly as possible, taking into account the health consequences.

The contributions of the German status report on climate change and health suggest options for action that are both relevant for climate protection and climate change adaptation and may also promote health and well-being. Since public health is not yet fully oriented towards climate change and its consequences [3], it seems appropriate to frame target parameters for Germany on the basis of these options for action. Based on a summary classification of the clusters of options for action (see Section 2 Options for action), target parameters for public health and related policy areas that can be implemented in the short to medium term are named below:

1. Further development of legal frameworks for coordinated structural and behavioural prevention in line with HiAP, especially in the sectors of health (e.g. public health service and health-related laws of the federal states), environment, climate, construction/housing, energy, and transport, as well as the institutional strengthening of cross-sectoral action
2. Provision of human and financial resources for the implementation of effective structural and behavioural prevention (e.g. in the areas of heat, UV prevention, pollen monitoring, and cross-sectoral cooperation with urban planning and building/housing)
3. Harmonisation of climate, environment, and health monitoring and strengthening of surveillance at the local, state, and federal levels
4. Research on the health impacts of climate change and the aspect of social equity
5. Provision of resources for research on and implementation of wide-ranging evidence-based and target group-oriented education, information, and communication measures
6. Provision of resources for multi-method research on the effectiveness and success factors of implemented measures in diverse societal settings
7. Financing of climate change adaptation and mitigation measures in the healthcare sector, including economic incentives for climate change adaptation and mitigation measures (e.g. digitalisation, energy retrofits, supply chains, and the reduction of pharmaceutical waste), and the provision of CO_2_ monitoring systems for healthcare facilities
8. Enhanced participation of affected and involved groups (including municipal actors, healthcare workers, associations of affected persons) in the planning, development, and implementation of measures

These options for action correspond at many points with recommendations formulated for HHAPs [36, 37] or various expert reports on the topic [7–9]. In a long-term perspective (‘imagination challenge’) and vision, it is advisable to continuously (re-)adjust prevention and health promotion with regard to the dimensions of climate change beyond the year 2025 as part of a learning system [7, 90]. This could include health-relevant and emission-intensive areas in nutrition, housing, work, and mobility through the lens of a HiAP-informed approach [7–9]. In the health-relevant area of housing and construction alone, the switch to a sustainable circular economy can mean the significant mitigation of CO_2_ while at the same time providing enormous co-benefits for health – for example in terms of indoor air quality, noise, and heat protection – according to a UBA report from 2023 [91]. Nevertheless, the co-benefit approach, which is also emphatically promoted in the most recent IPCC report (2023), has so far been insufficiently incorporated into administrative action [74].

## 5. Conclusion and outlook

The German status report on climate change and health has comprehensively summarised the multitude of climate impacts on health in the areas of infectious diseases, AMR, NCDs, and mental health. Due to the dynamics of climate change, possible adaptation effects, and unresolved research questions, an update of the report is recommended.

On the basis of the status report, systematically compiled target parameters and concrete options for action are available for public health to help manage transformations in climate protection and climate change adaptation that are oriented towards health promotion and social justice. Civil society movements and organisations, such as Fridays For Future, Health for Future, and Climate Alliance Germany, as well as many political and media debates in recent years, assess the climate protection measures taken so far as insufficient for achieving the agreed climate goals. The Federal Constitutional Court has supported this view in its climate protection decision (2021, BvR 2656/18). The majority of the population in Germany also evaluate the current measures for mitigating climate change effects as too weak and expect politicians to deliver cross-sectoral and cross-party solutions for greater climate protection [70].

In addition to individual motivation and willingness to change behaviour, which should simultaneously be healthier and climate-neutral, there is a compelling need for structural measures and policies that create appropriate conditions for the easier and more just implementation of climate protection and climate adaptation, and their integration into individual everyday practices.

Now and in the future, adaptation measures in climate protection and adaptation should be aligned with the concepts of health promotion, such as ‘health for all’, ‘health-related equal opportunities’, and ‘making healthy choices easy choices’. This requires cross-sectoral forums – in public health, medicine, urban and spatial planning, transport, and political contexts – that address conflicting goals and jointly work out co-benefits. The overarching goal is always to promote and accompany the concrete implementation of climate protection and climate change adaptation, in order to ensure that increased healthcare needs are met and that a climate-resilient healthcare system as well as climate protection and adaptation in line with HiAP become possible.

Ultimately, a societal subsystem, such as the field of public health, can only achieve an effective contribution to climate change mitigation and adaptation measures if it is embedded in overall societal and specifically economic policy transformation processes. Here, ecological limits and social issues play essential roles that need to be carefully weighed against the existing economic growth paradigm [2, 85, 92]. Cities in Europe and Germany have already started to anchor and implement climate change adaptation and partly also climate protection in their structures. Such municipal processes need to be strengthened by enabling cooperation between all sectors involved to solve cross-cutting tasks across departmental boundaries [7–9, 93, 94]. Communicating the positive health implications of climate action will play a central role in strengthening cooperation. Thus, in the end, the words of the leaders of public authorities in Germany involved in the status report must be earnestly reiterated – only ‘together we can counter the effects of climate change’ [93, P. 3].

## Key statement

- Structural and behavioural prevention related to climate change and health should be coordinated and implemented cross-sectorally.
- The monitoring and surveillance of climate change-associated health impacts should be strengthened and digital technologies and early warning systems should be increasingly used or developed.
- To protect the climate, the healthcare system must become ecologically sustainable and increase its own resilience to climate change impacts.
- Information, awareness raising, communication, and the participation of all stakeholders in the healthcare sector and beyond are important to implement health-related climate change mitigation and adaptation.
- Conflicting goals and resistance can be proactively addressed through participatory negotiation processes and by highlighting health-related co-benefits.
- Cross-sectoral activities should be strengthened so that frameworks can be developed for both structural and behavioural prevention within a ‘Health in All Policies’ approach.

## Figures and Tables

**Table 1:** Meta-levels of action and their guiding principles Source: Own representation

Meta-levels of action and their guiding principles
**a) Cross-sectorally coordinated structural and behavioural prevention** ‘Implementing cross-sectoral planning processes’ ‘Implementing urban (health) planning measures’ ‘Using governance for structural prevention’ ‘Using existing tools in development and planning’ ‘Promoting behavioural prevention through structures’
**b) Monitoring, surveillance, and digitalisation (including early warning systems)** 6. ‘Strengthening monitoring and surveillance’ 7. ‘Using digital technologies for climate adaptation and climate protection’
**c) Development of an ecologically sustainable and resilient public health system** 8. ‘Making the healthcare system sustainable and climatefriendly’
**d) Information, communication, and participation** 9. ‘Sharing knowledge and educating through participation’ 10. ‘Promoting target group-oriented awareness raising and social equity’
